# Effects of XPAND® on anaerobic power, muscular strength & subjective measures of mood state

**DOI:** 10.1186/1550-2783-10-S1-P9

**Published:** 2013-12-06

**Authors:** Sara Hayward, Stacie Urbina, Craig Jones, Josh Holt, Bailey Burks, Katilee Ralph, Cliffa Foster, Shawn Wells, Rob Wildman, Abbie Smith-Ryan, Lem Taylor, Colin Wilborn

**Affiliations:** 1 University of Mary Hardin-Baylor, Human Performance Lab, Belton, TX 76513, USA; 2 Dymatize Nutrition, Farmers Branch, TX 75234, USA; 3 University of North Carolina Chapel Hill, Depart of Exercise Science, Chapel Hill, NC, 27599, USA

## Background

Popular sports supplements contain a number of ingredients claiming to increase performance and enhance muscle gain. Product specific research is important for identifying efficacy of combined ingredients. The purpose of this study was to evaluate the effects of the proprietary pre-workout dietary supplement Dymatize XPAND, containing Creatine, CarnoSyn® Beta-Alanine, vitamins, L-Tarurine, L-Leucine, micronized pure and caffeine, on anaerobic power, muscular strength and endurance, body composition, as well as subjective measures of alertness, focus, energy, concentration, and hunger.

## Methods

In a double-blind, randomized ,matched -pair design, 12 males subjects (n = 12,mean ± SD; 22.4 ± 9.5 yrs, 171.3 ± 11.2 cm, 76.9 ± 11.2 kg, 22.7 ± 9.5% body fat), consumed either a 3 scoop serving of Dymatize XPAND (DX) or placebo (PLC) 30 minutes before completing a resistance training workout. Weight and body composition were determined via dual-energy x-ray absorptiometry (DEXA; Hologic Wi) after an 8 hour fast. Subjects then completed 12 vertical jumps for height (VJ), followed by 1 repetition maximum lifts on the bench press (MBP) and leg press (MLP). Muscular endurance for bench press (RBP) and leg press (RLP) was measured by completing as many repetitions as possible at 85% of the achieved MBP and MLP. Finally, the subjects completed a wingate power test on a cycle ergometer (insert manufacturer info) for measures of mean power (WMP) and peak power (WPP). The participants were then randomized into an eight day supplementation period with four resistance-training bouts spread over the eight days. Mood state and side effect questionnaires were completed each day after taking the supplement. After the supplementation period, the subjects returned to the lab to complete post-testing. All data were analyzed utilizing a 2 × 2 repeated measures ANOVA, treatment (PLC vs. DX) × time (pre-test vs. post-test) ANOVA. Ninety-five percent confidence intervals were also used. A Kruskal Wallis one-way analysis of variance was used for all survey data. A significance value of p<0.05 was adopted throughout.

## Results

There were no significant treatment × time interactions (p>0.05). There were no significant changes in %BF (Δ-.43±.58;p=0.920), FM (Δ-2.45±5.72;p=0.988), or LBM (10.9±12.2;p=848). 95% CI did demonstrate a significantly greater loss in %BF for the DX group. There was a main effect for WPP (Δ100.5 ± 42.7W; p=0.001), MBP (Δ8.0 ± 12.9 lbs; p=0.001), and MLP (Δ80.0 ± 28.8lbs; p=0.001), with no significant differences between treatments (p=0.138-0.253). There was no significant difference in mood states or appetites between the groups.

## Conclusion

The results of this study revealed that the proprietary blend Dymatize XPAND® may be effective, when combined with 8 days of training, for reducing %BF. While not significant, greater gains in MLP were demonstrated in the DX group. Future studies should evaluate more chronic effects of proprietary pre-workout blends on total training volume and performance outcomes.

